# *Pv*ML1 suppresses bacterial infection by recognizing LPS and regulating AMP expression in shrimp

**DOI:** 10.3389/fimmu.2022.1088862

**Published:** 2022-12-28

**Authors:** Yue Wang, Li-Guo Yang, Guang-Peng Feng, Zong-Li Yao, Shou-Hu Li, Jun-Fang Zhou, Wen-Hong Fang, Yi-Hong Chen, Xin-Cang Li

**Affiliations:** ^1^ Key Laboratory of Inland Saline-alkaline Aquaculture, Ministry of Agriculture and Rural Affairs, Shanghai, China; ^2^ East China Sea Fisheries Research Institute, Chinese Academy of Fishery Sciences, Shanghai, China; ^3^ Laboratory of Marine Biological Resources and Molecular Engineering, Marine Science and Technology College, Zhejiang Ocean University, Zhoushan, China; ^4^ Key Laboratory for Healthy and Safe Aquaculture, Institute of Modern Aquaculture Science and Engineering (IMASE), College of Life Science, South China Normal University, Guangzhou, China; ^5^ Southern Marine Science and Engineering Guangdong Laboratory, Zhuhai, China

**Keywords:** *Penaeus vannamei*, MD2-related lipid-recognition (ML) homologs, toll signaling pathway, antibacterial activity, LPS binding activity, recognition and activation mechanism

## Abstract

Toll and Toll-like receptors (TLRs) play essential roles in the innate immunity of *Drosophila* and mammals. Recent studies have revealed the presence of Toll-mediated immune signaling pathways in shrimp. However, the recognition and activation mechanism of Toll signaling pathways in crustaceans remain poorly understood due to the absence of key recognition molecules, such as peptidoglycan recognition proteins. Here, a novel MD2-related lipid-recognition (ML) member named *Pv*ML1 was characterized in *Penaeus vannamei*. We found that *Pv*ML1 shared a similar 3D structure with human MD2 that could specifically recognize lipopolysaccharides (LPS) participating in LPS-mediated TLR4 signaling. *PvML1* was highly expressed in hemocytes and remarkably upregulated after *Vibrio parahemolyticus* challenge. Furthermore, the binding and agglutinating assays showed that *Pv*ML1 possessed strong binding activities to LPS and its key portion lipid A as well as *Vibrio* cells, and the binding of *Pv*ML1 with bacterial cells led to the agglutination of bacteria, suggesting *Pv*ML1 may act as a potential pathogen recognition protein upon interaction with LPS. Besides, coating *V. parahemolyticus* with recombinant *Pv*ML1 promoted bacterial clearance *in vivo* and increased the survival rate of bacterium-challenged shrimp. This result was further confirmed by RNAi experiments. The knockdown of *PvML1* remarkably suppressed the clearance of bacteria in hemolymph and decreased the survival rate of infected shrimp. Meanwhile, the silencing of *PvML1* severely impaired the expression of a few antimicrobial peptides (AMPs). These results demonstrated the significant correlation of bacterial clearance mediated by *Pv*ML1 with the AMP expression. Interestingly, we found that *Pv*ML1 interacted with the extracellular region of *Pv*Toll2, which had been previously shown to participate in bacterial clearance by regulating AMP expression. Taken together, the proposed antibacterial model mediated by *Pv*ML1 might be described as follows. *Pv*ML1 acted as a potential recognition receptor for Gram-negative bacteria by binding to LPS, and then it activated *Pv*Toll2-mediated signaling pathway by interacting with *Pv*Toll2 to eliminate invading bacteria through producing specific AMPs. This study provided new insights into the recognition and activation mechanism of Toll signaling pathways of invertebrates and the defense functions of ML members.

## Introduction

Innate immunity is evolutionarily conserved and present in both invertebrates and vertebrates, and it plays a key role in the defense against invasions of a variety of pathogens (1, 2). In classical innate immune responses, pattern recognition receptors (PRRs) sense and specifically bind to the pathogen-associated molecular patterns (PAMPs) of invading pathogens, which results in activating innate immune responses to generate diverse immune effectors, thereby facilitating the elimination of the pathogens (3). Some PRRs, such as Toll-like receptors (TLRs), peptidoglycan recognition proteins (PGRPs), lipopolysaccharide (LPS) and β-glucan-binding proteins (LGBPs), are typical recognition proteins and regarded as “on and off” molecules for controlling the activation of individual signaling pathways (4–7).

MD-2-related lipid-recognition (ML) family proteins have diverse biological functions, but only a few family members possess immune recognition functions involved in host defense (8). ML proteins possess a putative *N*-terminal signal peptide and a ML domain at the *C*-terminus, and they can recognize a variety of lipids with ML domains (8, 9). ML domains have been identified in mammalian MD1, MD2, Niemann–Pick type C2 protein (NPC2), GM2 activator protein (GM2A), phosphatidylinositol/phosphatidylglycerol transfer protein (PG/PI TP), and mite allergen Der p 2 (8, 9). Human MD2 is a soluble endogenous ligand for TLR4 and a receptor for LPS (10). The LPS recognition and activation process of the TLR4 signaling pathway involve at least four different proteins (11). Among them, MD2 and TLR4 are the core components. MD2 specifically binds to LPS to form a ternary complex by interacting with the extracellular region of TLR4, subsequently activating this signaling pathway (12).

Toll and TLR-mediated signaling pathways play essential roles in the innate immune response of *Drosophila* and higher mammals, respectively (13, 14). Certain PGRPs act as PRRs in Toll signaling pathways by recognizing bacterial PAMPs, which triggers a humoral cascade of proteases leading to the activation of the pathway to produce AMPs and ultimately eliminate the intruders (15–17). In contrast to Tolls in *Drosophila*, TLRs in mammals directly bind to different PAMPs without the participation of PGRPs or with the assistance of accessory proteins, thereafter activating their respective signaling pathways (18–20).

Studies on the innate immunity of crustaceans, especially shrimp, have attracted widespread attention and achieved great progress in the past decade due to huge economic losses caused by a variety of pathogen infections (21–23). Most counterparts of the essential components in the Toll signaling pathway of *Drosophila* have been identified in shrimp, and current evidence even supports the existence of this pathway (19). However, the Toll signaling pathways of *Drosophila* and shrimp differ from each other, although they both belong to arthropods and share a close evolutionary relationship (19). A notable difference is the abundance of PGRPs in *Drosophila*, some of them even acting as “on and off” switches in Toll and IMD signaling pathways (14, 24), whereas they have not yet been reported in shrimp. Moreover, no PGRP gene homolog has been identified in the updated genome and transcriptome databases of crustaceans (shrimp and crab) (25–27). Thus, the recognition and activation mechanism of the Toll signaling pathway in shrimp remains unclear, and what strategy for activating the Toll signaling pathway in crustaceans needs further studies to clarify.

Recent studies have shown that only a few ML family members from crustaceans and insects are involved in immune responses. *Pj*ML1 could specifically bind to a lipid component (cholesta-3,5-diene) and initiate an anti-WSSV immune signaling pathway (28); two mud crab MD2 homologs recognized LPS and participated in anti-bacterial immunity (29, 30); and at least two insect ML members were involved in LPS signaling (31, 32). Considering that human MD2 is involved in LPS signaling (12), we speculate that certain ML homologs from crustaceans may similarly participate in the immune signaling pathways against Gram-negative bacteria. To verify this hypothesis, we characterized a novel ML homolog in *P. vannamei* (*Pv*ML1) and found that it could participate in the immune response against *V. parahemolyticus* by specifically binding to LPS. Furthermore, *Pv*ML1 could interact with the extracellular region of *Pv*Toll2, which mediates an immune signaling pathway (33). Taken together, our study was able to demonstrate the potential of *Pv*ML1 to act as a PRR or a co-receptor to participate in the antibacterial immune response of shrimp. This study provides new insights into the immune functions of ML members and the recognition and activation mechanisms of Toll signaling pathways in invertebrates.

## Materials and methods

### Reagents,chemicals and microorganisms

RNAiso Plus, First-Strand cDNA Synthesis Kit, *in vitro* Transcription T7 Kit and Taq Polymerase were purchased from TaKaRa Biotech (Dalian, China). Ultrapure LPS-EK (tlrl-peklps) were obtained from *In vivo*Gen. Lipoteichoic acid (LTA, from *Staphylococcus aureus*) were obtained from Sigma (St. Louis, MO, USA). *V. parahemolyticus* and *Vibrio harveyi* identified in our laboratory as well as four standard strains *Escherichia coli* (8099), *S*. *aureus* (ATCC 6538), *Bacillus megaterium* (NBRC 15308) and *Bacillus subtilis* (ATCC 9372) were used in this study.

### Tissue collection and immune challenge

Pacific white shrimp *P. vannamei* (~ 12 g each) were purchased from a shrimp farm in Ganyu County (Lianyungang, Jiangsu, China) were cultured in a cement tank with aerated seawater and fed daily with a commercial diet. The animal experiments were strictly conducted following the rules of the Institutional Animal Care and Use Committee of China. Healthy shrimp were randomly selected to analyze the tissue distribution and expression profiles of *PvML1*. Shrimp hemolymph was harvested with a sterilized syringe preloaded with ice-cold anticoagulant buffer (0.45 M NaCl, 0.1 M glucose, 30 mM trisodium citrate, 26 mM citric acid, and 10 mM ethylenediaminetetraacetic acid; pH 4.6), and then centrifuged at 850 × *g* for 15 min at 4°C to isolate hemocytes. Other tissues, including gills, hepatopancreas, intestine, heart, muscle, stomach, and eyestalk, were also dissected, washed with sterile PBS, and pooled from at least five healthy shrimp. All these tissues together with hemocytes were used to isolate total RNA for investigation of tissue distribution. For immune challenge, each shrimp was injected with 100 μL of bacterial inoculum (2 × 10^6^ CFU *V. parahemolyticus*). The corresponding control was treated with an equal volume of sterile phosphate-buffered saline (PBS) (140 mM NaCl and 10 mM sodium phosphate; pH 7.4). At each time point post injection (0, 3, 6, 12, 24 and 48 h), the total RNA of hemocytes was extracted for investigating the temporal expression pattern of *PvML1*. The extracted RNA was kept in 75% ethanol at −80°C until needed. Two other batches of RNA samples isolated previously were used to eliminate the differences among batches.

### Total RNA isolation and cDNA synthesis

RNAiso Plus reagent was used to extract the total RNA from hemocytes and other collected tissues. DNase I (Promega, USA) was added into the extracted RNA to remove contaminating genomic DNA. The cDNA was synthesized using the total RNA according to the manufacturer’s instructions of First-Strand cDNA Synthesis Kit.

### *Pv*ML1 cDNA cloning

The original cDNA sequence encoding the putative *Pv*ML1 was harvested through high-throughput transcriptome sequencing with an RNA mixture extracted from the hemocytes and hepatopancreas of shrimp. This cDNA sequence was further verified by polymerase chain reaction (PCR) with a pair of gene-specific primers (*Pv*ML1F and *Pv*ML1R, **Table 1**). The PCR was performed under the following parameters: 95°C for 3 min; 35 cycles of 94°C for 30 s, 54°C for 30 s, and 72°C for 30 s; and a final extension for 10 min at 72°C. The targeted DNA fragment was purified, cloned into a pMD-19T vector, and finally sequenced by Sangon Company (Shanghai, China).

**Table 1:** Sequences of primers used in this study.

Primers	Sequence (5′–3′)
cDNA cloning	
*Pv*ML1F	CCGGCGGGCACACTTAAA
*Pv*ML1R	GCGTGTGCGTGTGTGTGT
Real-time PCR	
*Pv*ML1RF	TTCACGCCAGACCGAAACCT
*Pv*ML1RR	ACGTCCCTCAGTCGCCAGAT
*Pv*EF1αF	GTATTGGAACAGTGCCCGTG
*Pv*EF1αR	ACCAGGGACAGCCTCAGTAAG
Protein expression	
*Pv*ML1EF	TACTCA ***GAATTC*** GAGGTGCACGAGATCCCCGT
*Pv*ML1ER	TACTCA ***CTCGAG*** TTACAAGATTTTAACATTGAAGACG
*Pv*Toll1EF	CGC ***GGATCC*** GTCACACTTTCTCTGTCTTG
*Pv*Toll1ER	TCC ***CCCGGG*** TCAGGGATTTCTGAATGAT
*Pv*Toll2EF	CGC ***GGATCC*** TTCAGCCCGTGTGGCAAG
*Pv*Toll2ER	TCC ***CCCGGG*** TCAGACCTCCGGCGGCAAAATAAT
RNAi	
*Pv*ML1iF	GCGTAATACGACTCACTATAGGGGGAGGTGCACGAGATCCCCGT
*Pv*ML1iR	GCGTAATACGACTCACTATAGGGGTTACAAGATTTTAACATTGAAGACG
EGFPiF	GCGTAATACGACTCACTATAGGGTGGTCCCAATTCTCGTGGAC
EGFPiR	GCGTAATACGACTCACTATAGGGCTTGAAGTTGACCTTGATGCC
AMPs	
*Pv*ALF1RF	TTACTTCAATGGCAGGATGTGG
*Pv*ALF1RR	GTCCTCCGTGATGAGATTACTCTG
*Pv*ALF2RF	GGCCATTGCGAACAAACTCAC
*Pv*ALF2RR	GTCCATCCTGGGCACCACAT
*Pv*ALF3RF	CTCCGTGTTGACAAGCCTGG
*Pv*ALF3RR	GCAGCTCCGTCTCCTCGTTC
*Pv*ALF4RF	ACCTGTCCAACCCTGAGCAAC
*Pv*ALF4RR	CCCTTTTCTACGACCTTCCTCAC
*Pv*PEN2RF	GACGGAGAAGACAATGGAAACC
*Pv*PEN2RR	ATCTTTAGCGATGGATAGACGAA
*Pv*PEN3RF	TACAACGGTTGCCCTGTCTCA
*Pv*PEN3RR	ACCGGAATATCCCTTTCCCAC
*Pv*PEN4RF	GGTGCGATGTATGCTACGGAA
*Pv*PEN4RR	CATCGTCTTCTCCATCAACCA
*Pv*Crus1RF	GTAGGTGTTGGTGGTGGTTTC
*Pv*Crus1RR	CTCGCAGCAGTAGGCTTGAC
*Pv*Crus2RF	GGTACGTCTGCTGCAAGCC
*Pv*Crus2RR	CTGAGAACCTGCCACGATGG
*Pv*Crus3RF	TCCACAATGGTCAGCGTCAAG
*Pv*Crus3RR	CTGTCCGACAAGCAGTTCCTC

### Bioinformatics analyses

The similarities of *Pv*ML1 with other ML family proteins were analyzed using the online Basic Local Alignment Search Tool Program (BLASTP) (http://blast.ncbi.nlm.nih.gov/Blast.cgi). The deduced protein sequences were translated and predicted on http://web.expasy.org/translate/. The putative domain was predicted using Simple Modular Architecture Research Tool (SMART) (http://smart.embl-heidelberg.de). Multiple alignment was conducted with the ClustalX 2.0 program (http://www.ebi.ac.uk/tools/clustalw2) and GENEDOC software. The theoretical molecular weight (Mw) and isoelectric point (pI) were calculated on http://web.expasy.org/compute_pi/. Signal peptide was searched with SignalP (34). A neighbor-joining phylogenetic tree was generated with MEGA 7.0 and 1000 bootstraps were used to assess reliability (35). Three-dimensional (3D) model of *Pv*ML1-lipid A complex was predicted by docking with BSP-SLIM ONLINE software (https://zhanglab.ccmb.med.umich.edu/BSP-SLIM/) and displayed by PyMOL program. The receptor protein *Pv*ML1 was modelled after the crystal structure of human MD2 (PDB ID: 2E59), and (heptosyl)2-Kdo2-lipid A was used as the ligand.

### Quantitative real-time PCR

qRT-PCR was carried out to analyze the mRNA expression levels of *Pv*ML1 and antimicrobial peptide (AMP) genes in a real-time thermal cycler Quantstudio 6 Flex (ABI, USA) following the protocol in a previous study (36). The gene-specific primers for *Pv*ML1 and AMP genes (**Table 1**) were designed to produce their respective amplicons and analyze their mRNA amounts. The primers for the internal reference gene *PvEF1α* (elongation factor 1-alpha, **Table 1**) were also synthesized and used to analyze the relative expression levels of *Pv*ML1 and AMP genes (37). qRT-PCR was performed in a 20-μL reaction mixture (10 μL of 2 × SYBR Premix Ex Taq, 2 μL of cDNA, and 4 μL of each primer). The reaction procedure was as follows: an initial denaturation step at 95°C for 3 min; 40 cycles at 95°C for 10 s, and 60°C for 40 s; and melting from 60°C to 95°C. The relative expression levels of *PvML1* in different tissues as well as AMP genes was calculated with the method of 2^−ΔCT^. The algorithm of 2^−ΔΔCT^ was applied to investigating the time-course profiles of *PvML1* (38). All treatments were carried out thrice with individual templates, and the obtained data were subjected to the statistical analysis. Significant differences were assessed by unpaired *t*-test (*P < 0.05; **P < 0.01).

### Recombinant expression and purification

Recombinant *Pv*ML1 as well as the extracellular regions of two *Pv*Tolls (*Pv*Toll1, ABK58729; *Pv*Toll2, AEK86516) was overexpressed with *E. coli* expression system. Based on *Pv*ML1 cDNA sequence, a pair of gene-specific primers (*Pv*ML1EF and *Pv*ML1ER, **Table 1**) were designed to amplify the DNA fragment (402 bp) encoding *Pv*ML1 mature peptide. After digestion with enzymes (*Eco*R I and *Xho* I), the fragment was ligated into a pET32a vector to construct recombinant plasmid pET32a-*PvML1*. Similarly, the DNA sequences encoding the extracellular regions of two Tolls were produced by PCR with two pairs of primers (**Table 1**); each fragment was digested by restricted enzymes and finally ligated into pGEX-6P-1 vectors. All these plasmids were respectively transformed into *E. coli* competent cells for over-expressions with isopropyl-β-d-thiogalactoside (IPTG, 0.1 mM). The recombinant *Pv*ML1 containing His tag was purified with Ni-NTA His Bind Resin, while the recombinant extracellular regions of Tolls (*Pv*Toll1ER and *Pv*Toll2ER) with GST tag were purified with glutathione sepharose 4B chromatography (Novagen, USA). Cold 0.1% Triton X-114 was used to remove contaminating endotoxins before collecting the final elution of the proteins from the column. Besides, the empty vectors pET32a and pGEX-6P-1 were also overexpressed in *E. coli*, and the corresponding vector proteins with thioredoxin (TRX) or GST tag were harvested and used as the negative controls.

### Microorganism-binding assay

Microorganisms, including Gram-negative bacteria (*V. parahemolyticus*, *V. harveyi* and *E*. *coli*) and Gram-positive bacteria (*S*. *aureus*, *B. megaterium* and *B. subtilis*), were applied to investigating the microorganism-binding activity of *Pv*ML1 using Western blot. The procedure was performed following our earlier study (39). Briefly, microorganisms were cultured in Luria–Bertani (LB) medium for 6 h at 37°C, and then were pelleted by centrifugation. After the pellets were washed thrice with 1 mL of TBS (50 mM Tris–HCl and 150 mM NaCl; pH 7.5), the microorganisms (1 × 10^8^ CFU) were incubated in 200 μL of r*Pv*ML1 (200 μg/mL) for 1 h at room temperature. Afterwards, they were pelleted, washed thrice with TBS, and eluted with 7% SDS by mild agitation for 5–10 min. The supernatants (eluates) were collected through centrifugation and the final pellets were harvested after three more washes with TBS. Both the eluates and the final pellets were subjected to 15% sodium dodecyl sulfate polyacrylamide gel electrophoresis (SDS-PAGE). r*Pv*ML1 was also sampled as the positive control. After separation with SDS-PAGE, the protein samples were transferred onto a nitrocellulose membrane. The membrane was blocked by 5% non-fat milk in TBS and then incubated with peroxidase-conjugated mouse monoclonal antibody against His-tag for 2 h. r*Pv*ML1 signal was visualized with an ECL Western blot detection reagent kit.

### Agglutination assay of *Pv*ML1

Gram-negative bacteria were chosen to investigate the agglutinating activity of *Pv*ML1. The agglutination assay was performed in accordance with the method described by Du. et al. (40). Bacteria cultured in LB broth were harvested at mid-logarithmic phase by centrifugation at 5000 × *g* for 5 min, washed three times with TBS, and then resuspended in TBS (2 × 10^8^ cells mL^–1^). The bacterial suspensions were incubated with equal volume (30 µL) of diluted r*Pv*ML1 in TBS at the protein concentration range of 0.8-5 µM with or without 10 mM CaCl_2_ at 28°C for 1 h. TRX tag protein (200 µg/mL) was used as the negative control. Agglutination was determined by observing under a light microscope. The minimal agglutinating concentration (MAC) is defined as the lowest protein concentration yielding visible microbial agglutination compared with the negative control.

### Enzyme-linked immunosorbent assay

ELISA was carried out to investigate the binding activities of *Pv*ML1 to microbial polysaccharides and *Pv*Tolls. Medium-binding microtiter plates (Greiner) were used to test the binding activity of *Pv*ML1 to microbial polysaccharides following a previous method (41). In brief, the plate wells were incubated with a total of 100 µL of LPS, Lipid A, or LTA (20 µg/mL) at 37°C overnight until the plate came to desiccation. Wells serving as the blank control were incubated with 100 µL of distilled water. After blocked with 200 μL of BSA (2 mg/mL) for 2 h and washed four times with TBST (0.05% Tween-20 in TBS), the wells were incubated with serially diluted recombinant *Pv*ML1 or TRX tag protein (negative control) (0.0005-1 μM in TBS containing 0.1 mg/mL BSA) at 37°C for 3 h and then rinsed five times with TBST. Each well was then incubated with 100 µL of peroxidase-conjugated mouse monoclonal anti-His antibody (1:5000 dilution in TBS with 1 mg/mL BSA). The color reaction was developed with 0.01% 3,3’,5,5’-tetramethylbenzidine (Sigma) and stopped with 2 M H_2_SO_4_. The absorbance was recorded at 405 nm by a microtiter plate reader (Tecan, Switzerland). In addition, high-binding microtiter plates (Greiner) were applied to investigating the binding function of *Pv*ML1 to *Pv*Tolls. The plates were pre-incubated with a total of 100 µL of r*Pv*toll1ER, r*Pv*toll2ER or GST (200 µg/mL) at 37°C for 2 h. After blocking with BSA and washing with TBST, serially diluted recombinant *Pv*ML1 or TRX tag protein (0.0005-1 μM in TBS containing 0.1 mg/mL BSA) was added to the plates. The color reaction was performed with the same procedure as the above, and the absorbance was obtained in the same way. All assays were performed in triplicate.

### RNA interference

A partial DNA fragment of *Pv*ML1 was amplified using primers containing a T7 promoter (*Pv*ML1iF and *Pv*ML1iR, **Table 1**). The harvested PCR product was used as the template to synthesize *dsPvML1* (*Pv*ML1 dsRNA) with an *in vitro* Transcription T7 Kit. The *dsEGFP* (EGFP dsRNA) was also synthesized as negative control with primers listed in **Table 1**. The healthy shrimp (~ 8 g each) were randomly divided into two groups (six shrimp in each group). Each shrimp was intramuscularly injected with 8 μg of *dsPvML1* or *dsEGFP* into the fourth abdominal segment. A second dsRNA injection was conducted 24 h later in the same manner. At 48 h after the first dsRNA injection, hemocytes was collected for total RNA extraction, which was used to assess RNAi efficiency by qRT-PCR. Experiments were performed independently thrice. Significant differences were analyzed with unpaired *t*-test (*P < 0.05; **P < 0.01).

### Bacteria clearance assay

After validating that *PvML1* expression could be silenced by injection of *dsPvML1*, we examined whether the knockdown of *PvML1* could affect bacterial clearance. *V*. *parahemolyticus* at the mid-logarithmic growth phase was collected by centrifugation and re-suspended in PBS (2 × 10^7^ CFU/mL) after washing three times. Each shrimp was injected with 100 μL of bacterial suspension at 48 h after injection with *dsPvML1* or *dsEGFP*. After mock injection with PBS, the shrimp were treated with an equal number of bacteria in the same way. At 40 min after bacterial injection, hemolymph (100 μL) was collected from shrimp and mixed with an equal volume of anticoagulant buffer. After serial dilution with PBS, the diluted hemolymph (50 μL) was smeared onto the LB plates. The plates were then incubated at 37°C until bacterial clones appeared. The number of residual bacteria in hemolymph was determined by counting the number of bacterial clones on the plates. In addition, to further confirm whether coating bacteria with *Pv*ML1 could facilitate bacterial clearance, *V. parahemolyticus* incubated with recombinant *Pv*ML1 or TRX tag protein was injected into shrimp following a method with slight modifications (42). Shrimp were randomly divided into two groups. Approximately 600 μL of r*Pv*ML1 or TRX tag protein in PBS (400 μg/mL) was mixed with an equal volume of bacterial suspension (2 × 10^7^ CFU/mL) with gentle rotation at room temperature for 15 min. TRX tag protein served as the control. After incubation, each shrimp was injected with 100 μL of mixture. The number of residual bacteria in hemolymph was calculated using the same method as described above. Unpaired student’s *t*-test was used to assess the significant differences. (*P < 0.05; **P < 0.01).

### Expression analysis of AMPs after *PvML1* knockdown

To investigate whether *PvML1* knockdown can affect the expression of AMPs in shrimp, ten different AMPs expressed in hemocytes were selected as representatives to assess the effectiveness caused by the decrease of *PvML1* expression. These AMPs are from three different AMP families: anti-lipopolysaccharide factors (ALFs), penaeidins (PENs) and crustins (Crus) (43). At 48 h after dsRNA (*dsPvML1* or *dsEGFP*) injection, the total RNAs of hemocytes were extracted, and cDNAs were synthesized as the templates for qRT-PCR. The gene-specific primers for AMPs were listed in **Table 1**. Unpaired *t*-test was used to analyze significant differences (*P < 0.05; **P < 0.01).

### Analysis of survival rates

Survival rate assay was conducted to investigate the effect of *Pv*ML1 knockdown on host antibacterial immunity. Shrimp (~ 8 g each) were infected with 100 μL of *V. parahemolyticus*(1 × 10^7^ CFU)at 48 h after first dsRNA (*dsPvML1* or *dsEGFP*) injection. Shrimp received two times of PBS injection served as blank control. The numbers of dead animals were recorded from 3 h to 24 h after bacterial injection, by which the survival percentage was determined. In addition, a total volume of 100 μL *V. parahemolyticus* (1 × 10^7^ CFU) pre-incubated with r*Pv*ML1 or with TRX tag protein in PBS was injected into shrimp to calculate the survival rates. Blank control was treated with an equal volume of PBS. A total of 30 shrimp was randomly selected for each group. The statistical analysis was conducted using Log-rank (Mantel-Cox) test.

### GST pull-down assay

Two *Pv*Tolls (*Pv*Toll1 and *Pv*Toll2) with higher similarities to human TLR4 were chosen to analyze the potential interactions between *Pv*ML1 and *Pv*Tolls by conducting GST pull-down assays according to a documented method with slight modifications (40). A total of 150 μL glutathione-Sepharose 4B resin (50% bead slurry) after wash three times with PBS was incubated with a mixture of a His-tagged protein (15 μg, r*Pv*ML1) and a GST-tagged protein (15 μg, r*Pv*Toll1ER, r*Pv*Toll2ER or GST) for 2 h at 4°C. The GST tag protein served as negative control. After incubation, the beads were washed thoroughly with PBS, and then proteins were eluted by adding PBS containing 10 mM reduced glutathione. The final washes and resultant eluates as well as the recombinant proteins, including r*Pv*ML1, r*Pv*Toll1ER and r*Pv*Toll2ER, were subjected to a 12.5% SDS-PAGE. The results were analyzed after the gel was stained with Coomassie blue.

### Plasmid constructions and co-immunoprecipitation assays

Based on the cDNA sequences of *Pv*Toll1 and *Pv*Toll2, two pairs of gene-specific primers (**Table 1**) were designed to amplify the DNA sequences of the extracellular region of these two *Pv*Tolls. Either the harvested DNA fragments or pcDNA3.1-myc-his-A (pcDNA3.1) vector were digested, and the targeted fragments were then ligated into a pcDNA3.1 vector to generate expression plasmids with the sequences of truncated *Pv*Tolls (pcDNA3.1-*Pv*Toll1ER and pcDNA3.1-*Pv*Toll2ER). Besides, the DNA sequence encoding the mature peptide of *Pv*ML1 was amplified with the specific primers in **Table 1**, and then ligated into pcDNA3.1-EGFP to produce a recombinant plasmid pcDNA3.1-*Pv*ML1-EGFP. HEK 293T cells were cultured in high-glucose DMEM medium (Gibco) supplemented with 10% fetal bovine serum (FBS), 100 U/ml penicillin and 100 µg/ml streptomycin, in humidified 5% CO_2_ and 95% air at 37 °C. For transient transfection, cells were seeded into 6-well microtiter plates and incubated overnight. When cells were ~ 70% confluent, the cells were co-transfected with 2 μg of His-tagged expression plasmid (pcDNA3.1-*Pv*Toll1ER or pcDNA3.1-*Pv*Toll2ER) and 2 μg of EGFP-tagged expression plasmid pcDNA3.1-*Pv*ML1-EGFP. At 36 h after transfection, the cells were lysed with NP40 lysate (Beyotime) and then centrifuged at 12,000 rpm for 20 min at 4°C, and the supernatants were incubated with anti-cGFP antibody (or anti-cMyc antibody) and Protein A + G Agarose beads overnight at 4°C with rotation. Normal rabbit IgG was used as the negative control. The beads were collected by centrifugation, washed three times with PBS, and then resuspended in 1 × SDS sample buffer. After boiling for 10 min, the resultant samples were separated by SDS-PAGE and then were analyzed by Western blot.

## Results

### Nucleotide and amino acid sequences of *Pv*ML1

**Figure 1:**
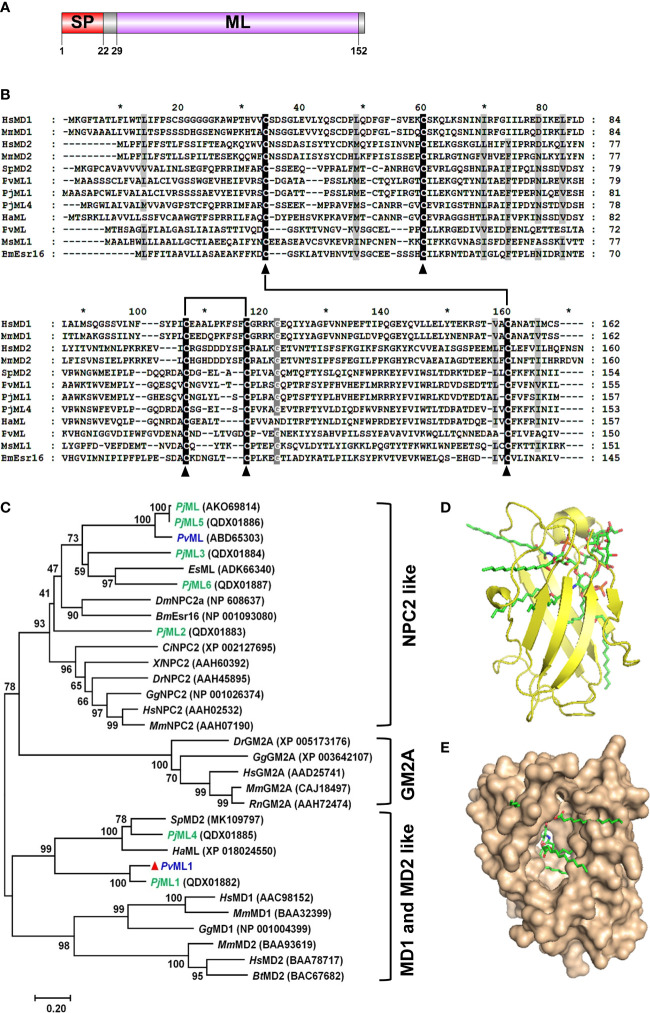
Sequence and architecture information of *Pv*ML1. **(A)** Schematic of *Pv*ML1 domains was predicted with SMART software. **(B)** Multiple alignment of *Pv*ML1 with other representative ML domain-containing proteins. *Hs*, *Homo sapiens*; *Mm*, *Mus musculus*; *Sp*, *Scylla paramamosain*; *Pv*, *Penaeus vannamei*; *Pj*, *Penaeus japonicus*; *Ha, Hyalella azteca*; *Ms, Manduca sexta*; *Bm, Bombyx mori*. **(C)** Phylogenetic analysis of *Pv*ML1 and other retrieved ML domain-containing proteins by MEGA 7.0. Bootstrap values were showed at each node, and *Pv*ML1 was marked with red triangle. ML protein family from *P*. *japonicus* was highlighted in green. ML protein of *P*. *vannamei* were highlighted in blue. The corresponding GenBank accession numbers and names were listed. *Es*, *Eriocheir sinensis*; *Dm*, *Drosophila melanogaster*; *Ci*, *Ciona intestinalis*, *Dr*, *Danio rerio*; *Gg*, *Gallus gallus*; *Rn, Rattus norvegicus*. The predicted 3D model of *Pv*ML1-Lipid A complex. The *Pv*ML1-Lipid A complex with a docking score of 5.981 was shown in two different manners (observe from the side **(D)** or from the opening of the protein ‘cavity’ **(E)**). The ligand lipid A was displayed in stick.

The complete cDNA sequence of *Pv*ML1 had 652 bp, including a 144-bp 5′ untranslated region, a 468-bp open reading frame for encoding a 155-amino acid (aa) polypeptide, and a 3′ noncoding region of 40 bp (GenBank Accession No. MN604018) (**Figure S1**). A signal peptide of 22 residues at the *N*-terminus and a ML domain (29–152 aa) were found in deduced protein. The ML domain contained six conserved cysteines that may form three disulfide bonds to stabilize the overall structure. The domain architecture of *Pv*ML1 was schematically shown in **Figure 1A**. The mature peptide of *Pv*ML1 had an estimated Mw of 15.5 kDa and a theoretical pI of 7.85.

### Similarities and phylogenetic analyses

BLASTP search analysis showed that *Pv*ML1 shared the highest similarity (78.34%) with an ML domain-containing protein *Penaeus japonicus Pj*ML1 (QDX01882), but the similarity did not go beyond 40% with other ML proteins. For instance, *Pv*ML1 had 32.69% similarity with *Scylla paramamosain Sp*MD2 (MK109797), 30.97% with *Pj*ML4 (QDX01885), 16.03% with *P. vannamei Pv*ML (ABD65303), and 15.85% with *Homo sapiens* MD2 (BAA78717). The similarities among ML proteins were further revealed by the alignment of representative ML protein sequences from different species. However, low similarities were observed among the ML protein sequences, except for the five highly conserved cysteine residues present in each ML protein (**Figure 1B**). Earlier studies revealed that four of the five cysteine residues located in similar positions of ML proteins could form two disulfide bonds, which were responsible for maintaining the overall structure and biological functions of the ML proteins. *Pv*ML1 and other representative ML proteins presented four cysteine residues that were involved in disulfide-bond formation, suggesting that these ML proteins may have a similar 3D structure.

The evolutionary relationship between *Pv*ML1 and other ML proteins was analyzed by constructing a phylogenetic tree. In this tree, the vertebrate ML proteins were separated into three different meaningful clusters: NPC2, GM2A, and MD2 and MD1. The crustacean ML proteins were grouped into two of the three aforementioned clusters (**Figure 1C**). *Pv*ML1, *Pj*ML1, *Pj*ML4, *Sp*MD2, and *Ha*ML were clustered together with the vertebrate MD2 and MD1 homologs, whereas the other crustacean ML homologs were grouped together with the NPC2 homologs. *Pv*ML1 presented a much closer phylogenetic relationship with the vertebrate MD2 homologs, suggesting that it may possess similar immune function to human MD2 because the latter is an essential immune component of the human TLR4 signaling pathway.

### Lipid A was docked well with *Pv*ML1

Docking was performed with the receptor protein *Pv*ML1 and the ligand of lipid A (lipid portion of LPS) to determine whether *Pv*ML1 possessed LPS-binding potentials. The 3D model of the *Pv*ML1–lipid A complex with the highest docking score (5.981) was shown in **Figures 1D, E**. The *Pv*ML1 molecule contained a deep hydrophobic cavity, and lipid A was properly accommodated in it. This formation was similar to the *Hs*MD2–lipid A complex, which attained a slightly higher docking score of 7.510 (not shown).

### *Pv*ML1 was highly expressed in hemocytes and upregulated by bacterial challenge

**Figure 2:**
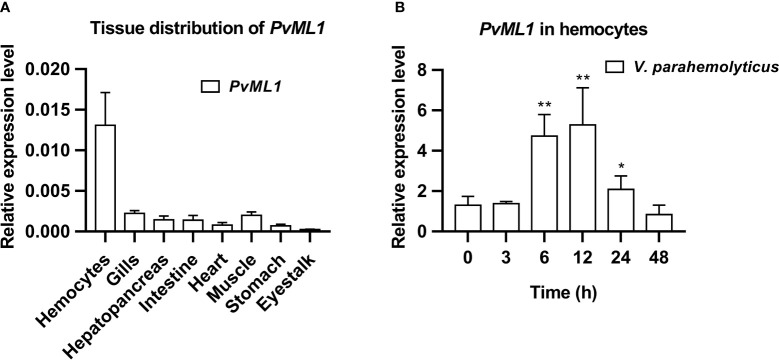
Tissue distribution and expression profiles of *PvML1*. **(A)** Tissue distribution of *PvML1* was analyzed using qRT-PCR with *EF1α* as the internal reference. **(B)** Expression profiles of *PvML1* in hemocytes at different time points after *Vibrio parahemolyticus* infection. Significant differences were indicated with asterisks (*P < 0.05; **P < 0.01).

qRT-PCR was conducted to investigate the tissue distribution and time-course expression profile of *PvML1* after bacterial challenge. As shown in **Figure 2A**, *PvML1* was expressed in all tested tissues, and its relative expression level in hemocytes was much higher than those in the other tissues (gills, hepatopancreas, intestine, heart, muscle, stomach, and eyestalk). *PvML1* was highly expressed in hemocytes, suggesting its important role in the open circulating system of shrimp. Then, the temporal expression profile of *PvML1* in hemocytes after the bacterial challenge was further investigated. *PvML1* was significantly increased 6–24 h after it was challenged with *V. parahemolyticus* and reached the highest expression level (nearly a sixfold increase) at 12 h post-injection (**Figure 2B**). This result revealed that *Pv*ML1 was an immune component involved in the antibacterial response of shrimp.

### Recombinant proteins were successfully expressed and purified

**Figure 3:**
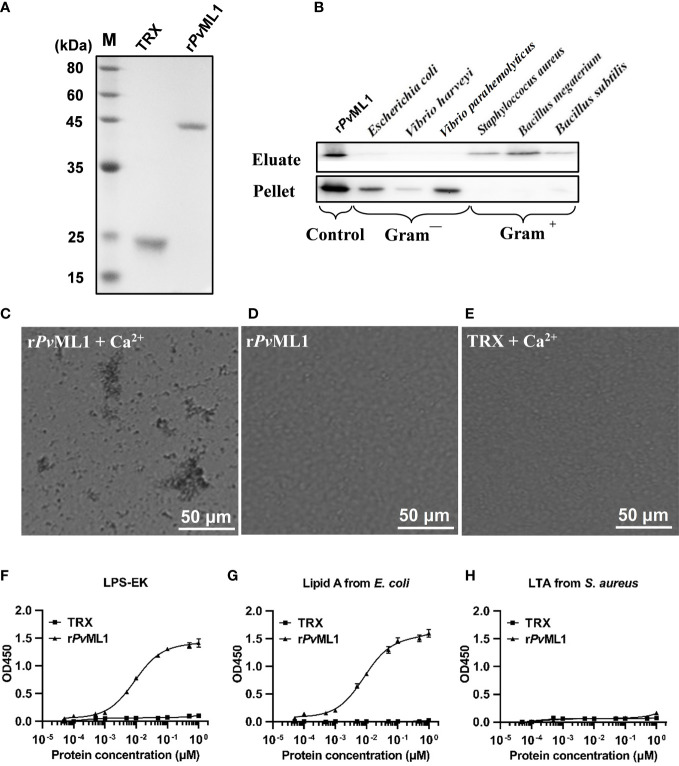
*Pv*ML1 possessed a strong binding and agglutination activity to *Vibrio* cells by binding to Lipid A of LPS. **(A)** Recombinant *Pv*ML1 (r*Pv*ML1) and TRX were expressed in *E. coli* and then purified. Lane M, protein marker; Lane TRX, the purified tag protein TRX; Lane r*Pv*ML1, the purified recombinant protein *Pv*ML1. **(B)** Binding activity of *Pv*ML1 to different microorganisms. The binding activities of *Pv*ML1 were confirmed by Western blot. Eluate panel, elution fractions; Pellet panel, final pellet fractions. r*Pv*ML1 were sampled as the positive controls. Agglutination of *V. parahemolyticus* induced by *Pv*ML1. *V. parahemolyticus* was incubated with r*Pv*ML1 with **(C)** or without Ca^2+^ **(D)**. TRX was used as the negative control **(E)**. Agglutination was observed under light microscopy. Microbial polysaccharide-binding activities were investigated using ELISA. LPS-EK **(F)** and Lipid A from *E. coli* **(G)**, and LTA from *S. aureus* **(H)** were used to coat plates. r*Pv*ML1 and TRX (negative control) were serially diluted and then added to the polysaccharide-coated plates. Results were obtained from three independent experiments.

*Pv*ML1 and two truncated *Pv*Tolls were successfully expressed and purified. The recombinant *Pv*ML1 was expressed as a TRX-tagged fusion protein with a predicted Mw of 34.5 kDa (including the ~19 kDa TRX tag). Meanwhile, r*Pv*Toll1ER and r*Pv*Toll2ER were GST-tagged fusion proteins with predicted Mw values of 104.2 and 113.3, respectively (including the ~26 kDa GST tag). The position of each purified protein was roughly in agreement with the Mw of the corresponding recombinant protein (**Figure 3A**).

### *Pv*ML1 exhibited microbe-binding activity and agglutinated gram-negative bacteria

Western blot was performed to examine the microbial cell-binding ability of *Pv*ML1. r*Pv*ML1 was detected only in the eluate, suggesting its weak binding to the microorganisms. However, this protein was found in pellets, indicating strong binding ability. According to this standard, *Pv*ML1 exhibited a strong binding activity to Gram-negative bacteria (*V. harveyi*, *V. parahemolyticus*, and *E. coli*) (**Figure 3B**). *Pv*ML1 also displayed weak binding to other tested microorganisms (*S. aureus*, *B. subtilis*, and *B. megaterium*). The results suggest that *Pv*ML1 may act as a potential recognition protein for certain kinds of pathogens, especially Gram-negative bacteria. Then, the agglutination activities of *Pv*ML1 to microbes were investigated, especially since some immune components agglutinate pathogens *via* their microbial cell-binding activities. *Pv*ML1 exhibited remarkable agglutination to *V. parahemolyticus* cells in the presence of Ca^2+^ (**Figures 3C–E**). It also displayed agglutinating activities to *E. coli* and *V. harveyi*, and the agglutinating activity to the former is much stronger than the latter (**Table 2**). These results further demonstrated that *Pv*ML1 could specifically interact with certain components on the surface of Gram-negative bacteria.

**Table 2:** Agglutinating activity of *Pv*ML1.

Microorganisms	MAC (μM)	
	r*Pv*ML1 + Ca^2+^	r*Pv*ML1
Gram^−^		
*V*. *parahemolyticus*	< 0.31	^−^
*V*. *harveyi*	< 0.31	^−^
*E*. *coli*	< 0.08	^−^

Minimum agglutinating concentration (MAC) is defined as the lowest protein concentration harvesting significant agglutination compared with the negative control. ‘^−^’ means no significant agglutination was observed with the protein concentration of 5 μM.

### *Pv*ML1 exhibited strong binding activity to LPS

Considering that *Pv*ML1 could bind to the aforementioned microbes, certain components on the microbial cell surface might be recognized by *Pv*ML1. Furthermore, as most ML family members were determined as lipid-binding proteins, the common bacterial PAMPs with a lipid portion, such as LPS and its lipid portion (lipid A) and LTA, were selected and then applied to an ELISA. As shown in **Figures 3F–H**, r*Pv*ML1 could bind to both LPS and lipid A in a concentration-dependent manner within a certain concentration range. However, r*Pv*ML1 did not exhibit a significant binding activity to LTA. By contrast, the TRX tag protein exhibited much lower binding activities to both LPS and lipid A, although it could also interact with them. These results revealed the specific binding activity of *Pv*ML1 to LPS and lipid A, and its binding activity to LPS was largely contributed by the binding to lipid A. Thus, we speculate that LPS may be the key recognition site on the surface of Gram-negative bacteria, which can be sensed by binding to lipid A.

### Pre-incubating bacteria with r*Pv*ML1 Increased survival rate of shrimp by promoting bacterial clearance in hemolymph

**Figure 4:**
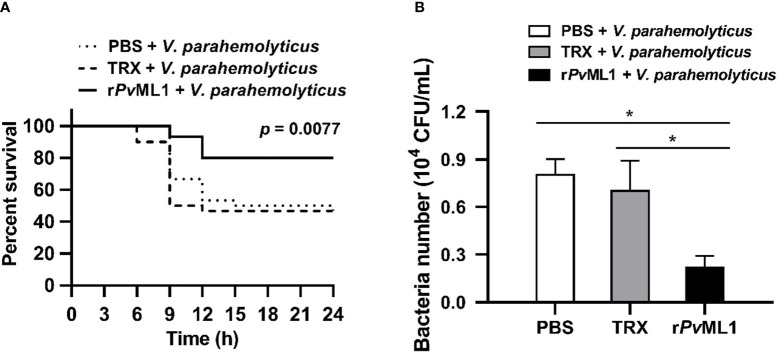
Protective role of *Pv*ML1 against bacterial infection and its effect on bacterial clearance in hemolymph. **(A)** *V. parahemolyticus* pre-incubated with r*Pv*ML1 was injected into shrimp to calculate the survival rates. Thirty shrimp were used for each group, and the results were analyzed by Log-rank (Mantel-Cox) test. **(B)** The ability to clear *V. parahemolyticus* in hemolymph was increased by the “overexpression” of *Pv*ML1 protein. TRX served as negative control, PBS was used as blank control (*P < 0.05).

Survival assay was performed to investigate the *in vivo* function of *Pv*ML1 by using *V. parahemolyticus* cells that were pre-incubated with r*Pv*ML1 or TRX protein. After the bacterial cells were injected into shrimp, the r*Pv*ML1 significantly enhanced the shrimp resistance against bacterial infection. As shown in **Figure 4A**, the survival rate of the r*Pv*ML1-treated group was always higher than that of the control group from 6 h after bacterial injection. The survival percentage of the control group was approximately 50% at 15 h after infection, whereas more than 70% shrimp were alive in the experimental group at that time. The abovementioned results confirmed the role of *Pv*ML1 in host immunity to protect shrimp from bacterial infection. To further explore whether *Pv*ML1 could facilitate bacteria clearance. *V. parahemolyticus* cells pre-incubated with r*Pv*ML1 or TRX protein were injected into healthy shrimp. In contrast to the findings involving TRX treatment, the number of bacteria in the hemolymph was significantly decreased 40 min after the injection with r*Pv*ML1-incubated bacteria, demonstrating that pre-incubating bacteria with r*Pv*ML1 could facilitate bacterial clearance *in vivo* (**Figure 4B**). Taken together, these results indicate that the increased survival rate of shrimp may be attributed to the promoted bacterial clearance in hemolymph by *Pv*ML1.

### *PvML1* knockdown decreased survival rate of shrimp

**Figure 5:**
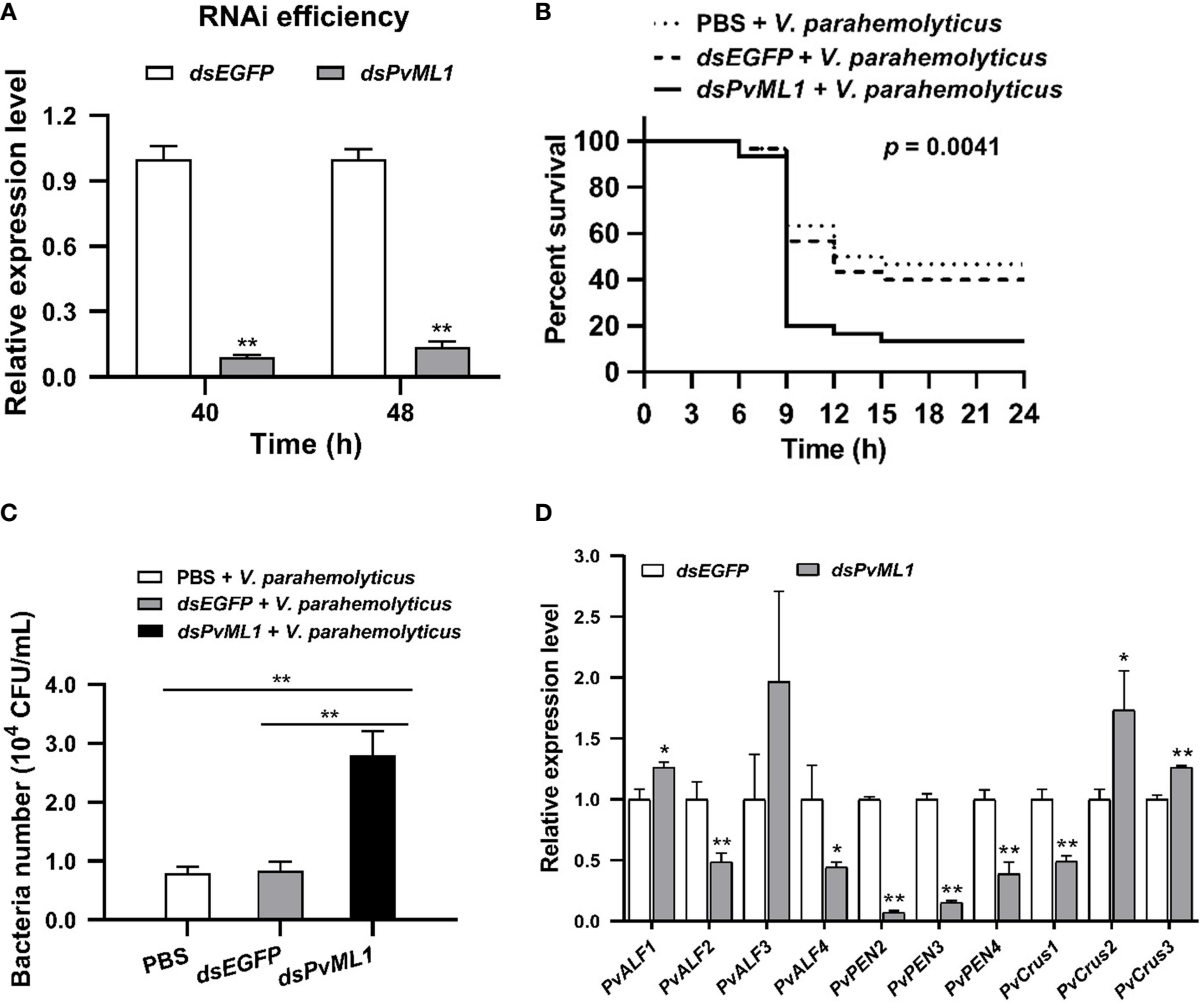
The effects by *PvML1* knockdown on survival rate, bacterial clearance, and AMP expression in hemolymph. **(A)** Effective knockdown for *PvML1* in hemocytes by dsRNA was confirmed by qRT-PCR. **(B)** Survival of *V. parahemolyticus* challenged *PvML1*-silenced shrimp and *EGFP* dsRNA treated shrimp. PBS was used as control. Thirty shrimp were used for each group, and the results were analyzed by Log-rank (Mantel-Cox) test. **(C)** Bacteria clearance experiment upon RNA interference with *dsEGFP* or *dsPvML1*. PBS was used as control. **(D)** qRT-PCR analysis of the downstream antimicrobial peptide genes. The results presented the mean of three individual experiments. Asterisks indicate the significant differences compared with values of the control (*P < 0.05; **P < 0.01).

RNAi of *PvML1* and survival assays were conducted to investigate the *in vivo* function of *Pv*ML1. qRT-PCR analysis showed a considerable downregulation of the expression level of *PvML1* in the hemocytes 40 and 48 h after the first injection of *dsPvML1*, whereas those of the remaining transcripts of *PvML1* in the *dsPvML1-*injected group did not exceed 20% of those in the control group at each time point (**Figure 5A**). This result indicated that injecting *dsPvML1* into shrimp could dramatically suppress *PvML1* expression. After *PvML1* knockdown, *V. parahemolyticus* was injected into shrimp, and the survival rates in different groups were calculated. In this manner, the antibacterial ability could be evaluated. As shown in **Figure 5B**, knockdown of *PvML1* dramatically suppressed host’s immune function against bacteria. The survival percentage of *dsPvML1*-treated shrimp 15 h after bacterial infection did not exceed 20% in the experimental group, whereas approximately 50% of *dsEGFP*-treated shrimp was still alive in the control group. This result suggests that *Pv*ML1 may function as an important antibacterial component in shrimp.

### Knockdown of *Pv*ML1 suppressed bacterial clearance in hemolymph

Bacterial clearance assays were conducted after *PvML1* knockdown to investigate the immune function of *Pv*ML1. After validating that *PvML1* expression could be knocked down, *V. parahemolyticus* was injected into the dsRNA-treated shrimp. Then, the residual bacterial number in the hemolymph was counted to determine the bacterial clearance ability. As shown in **Figure 5C**, the number of residual bacteria in hemolymph significantly increased 40 min post-injection compared with that in the *dsEGFP-*treated group. The results showed that *PvML1* knockdown remarkably suppressed bacterial clearance.

### *PvML1* knockdown significantly suppressed the expression of AMPs

Aimed at determining whether the presence of AMPs in the hemolymph were relevant to bacterial clearance, the expression level of AMPs in the hemocytes of shrimp 48 h after injection with *dsPvML1* or *dsEGFP* were investigated *via* qRT-PCR. The results showed the transcripts of *PvALF2*, *PvALF4*, *PvPEN2*, *PvPEN3*, *PvPEN4*, and *PvCrus1* were significantly decreased in *PvML1*-silenced shrimps compared with those in the control group. *PvALF1*, *PvCrus2*, and *PvCrus3* were significantly increased, and no significant changes were observed in the expressions of *PvALF3* (**Figure 5D**). These results demonstrated that *PvML1* knockdown significantly suppressed the expression of certain AMPs. The combined results suggested that the low expression of certain AMPs may be responsible for the decreased bacterial clearance ability caused by *PvML1* knockdown.

### *Pv*ML1 interacted with *Pv*Toll2 as well as *Pv*Toll1

**Figure 6:**
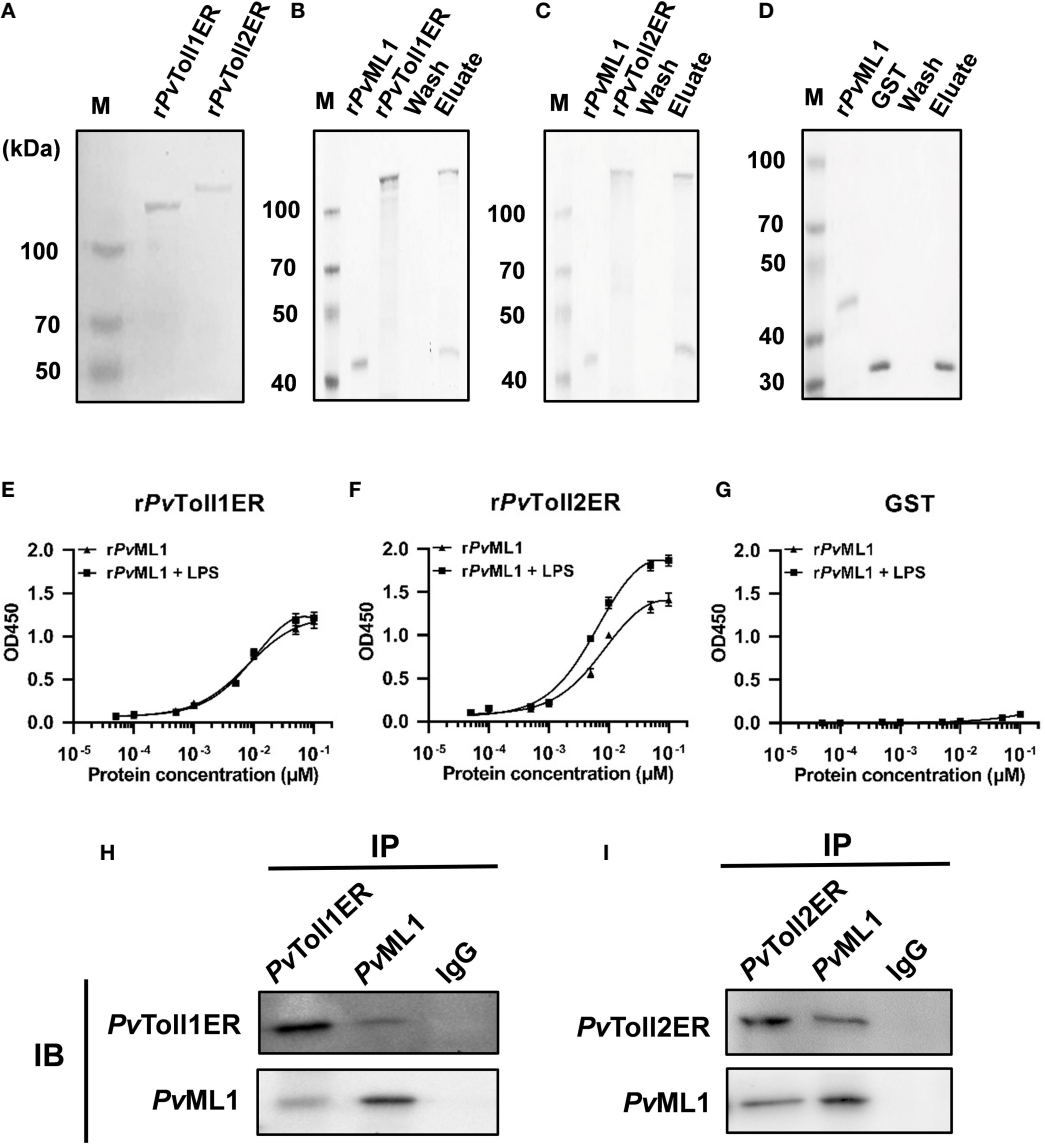
*Pv*ML1 interacted with the extracellular region of *Pv*Tolls. **(A)** Recombinant expression and purification of the extracellular region of *Pv*Toll1 and *Pv*Toll2. *Pv*Toll1ER and *Pv*Toll2ER were expressed with pGEX-6P-1 vector in *E. coli* Rosseta (DE3) cells and purified. **(B-D)** GST pull-down assay was carried out to test the interaction of *Pv*ML1 with *Pv*Tolls. r*Pv*ML1 and *Pv*TollERs (r*Pv*Toll1ER or r*Pv*Toll2ER) were mixed with Glutathione Sepharose 4B resin, and GST was used as control in this experiment. The results were visualized by coomassie blue staining. *Pv*ML1 interacted with GST-tagged *Pv*Toll1ER and *Pv*Toll2ER but not with GST. **(E-G)** ELISA was performed to analyze the binding ability of *Pv*TollERs to *Pv*ML1. r*Pv*Toll1ER, r*Pv*Toll2ER, or GST was used to coat plates. r*Pv*ML1, or r*Pv*ML1 plus LPS were serially diluted and added into the coated plates. **(H-I)** A Co-IP assay was performed to confirm the interaction between *Pv*TollERs and *Pv*ML1 in cells. Myc-tagged expression plasmid (pcDNA3.1-*Pv*Toll1ER, or pcDNA3.1-*Pv*Toll2ER) and EGFP-tagged expression plasmid (pcDNA3.1-*Pv*ML1-EGFP) were co-transfection into HEK-293T cells, respectively. Anti-cMyc antibody and anti*-*cGFP antibody were used to analyze the interaction. Normal rabbit IgG was used as the negative control.

To investigate whether *Pv*ML1 could interact with certain Toll homologs similar to human MD2 in the TLR4 signaling pathway, r*Pv*ML1 and r*Pv*Toll1ER and r*Pv*Toll2ER were prepared for the GST-pull down assay. The results were shown in **Figures 6B–D**. r*Pv*ML1 displayed apparent binding activities to both r*Pv*Toll1ER and r*Pv*Toll2ER but not to the GST tag protein. The interaction of r*Pv*ML1 with both r*Pv*Toll1ER and r*Pv*Toll2ER were further verified by ELISA (**Figures 6E–G**). The r*Pv*ML1 exhibited strong binding abilities to both r*Pv*Toll2ER and r*Pv*Toll1ER, and the binding activity of r*Pv*ML1 to r*Pv*Toll2ER was slightly stronger than that to r*Pv*Toll1ER. Furthermore, r*Pv*ML1 pre-incubated with LPS had a stronger binding activity to r*Pv*Toll2ER than that to r*Pv*Toll1ER. Besides conducting interaction assays with recombinant proteins, a co-immunoprecipitation assay was also performed by co-infecting *PvML1* and the truncated *PvTolls* into HEK-293T cells (**Figures 6H, I**). We found that *Pv*ML1 specifically interacted with *Pv*Toll2ER as well as *Pv*Toll1ER. These results suggest that *Pv*ML1 may act as an accessory recognition protein for LPS in *Pv*Toll2 signaling pathway.

## Discussion

PRRs play a key role in innate immunity by recognizing invading pathogens and mediating the activation of specific immune responses (3–6). Here, we identified a novel ML homolog in *P. vannamei* named *Pv*ML1. *Pv*ML1 displayed remarkable binding activities to LPS and lipid A and facilitated bacterial clearance by regulating the expression of specific AMPs in shrimp. In addition, *Pv*ML1 specifically interacted with the extracellular region of *Pv*Toll2. These findings suggest that *Pv*ML1 may be an upstream PRR for the *Pv*Toll2-mediated signaling pathway, and their interaction may facilitate the activation of *Pv*Toll2 signaling pathway to produce AMPs defending shrimp against the bacterial invasion.

More than one ML family member has been found in a few invertebrate species. For example, the *Drosophila melanogaster* genome encodes 8 ML family members, the *Anopheles gambiae* genome encodes 13 ML proteins, and 6 ML proteins have been identified in *P. japonicus* (28, 31, 44). However, the biological functions of these invertebrate ML homologs remain largely unknown. A recent report has shown that *Pj*ML1 from *P. japonicus* recognizes a lipid component of WSSV envelope participating in antiviral immune response (28). The authors also found that *Pj*ML1 and *Pj*ML4 are clustered with human MD2, whereas the other *Pj*ML homologs have a far evolutionary relationship with human MD2. Interestingly, *Sp*MD2, a crab ML homolog, participates in the immune response against Gram-negative bacteria by recognizing LPS, and it shares a close evolutionary relationship with human MD2 and *Pj*ML1 (29). These findings clearly demonstrate the involvement of some ML family members in immune responses in different ways. To date, only two *Pv*ML members (*Pv*ML and *Pv*ML1) have been identified in *P. vannamei*. Our current study showed that *Pv*ML1, *Pj*ML1, *Sp*MD2, and *Hs*MD2 were clustered into a large group, but *Pv*ML was disassociated to this group, displaying a distant evolutionary relationship with these molecules. Considering that *Pv*ML1 also shared a similar 3D structure with human MD2 and exhibited specific binding activities to LPS and lipid A, we speculate that *Pv*ML1 may be the homolog of human MD2. In fact, our study on the tissue distribution of *Pv*ML1 also revealed the more prominent similarity of *Pv*ML1 to the MD2 homolog compared with that to *Pv*ML. Similar to human MD2, which is widely present in the human fluid environment, further playing an important role in humoral immunity (10), *Pv*ML1 is a secreted protein that is highly expressed in hemocytes, and it participates in antibacterial infection in hemolymph. By contrast, *Pv*ML (*Lv*ML) is only highly expressed in the hepatopancreas, which is somewhat different from the tissue distribution profile of human MD2 (9, 10). As most members of the ML family participate in lipid metabolism, and because hepatopancreas is rich in lipid components, *Pv*ML may play an important role in certain lipid metabolism processes, although it has been shown to bind LPS (9). Taken together, *Pv*ML1 has a closer evolutionary relationship and a similar tissue distribution pattern with human MD2, and it carries out remarkable antibacterial activity by recognizing LPS, further suggesting that *Pv*ML1 may be the homolog of human MD2 in shrimp.

ML family members exhibit diverse biological functions by binding different lipid components with their ML domains (8). A typical ML domain consists of two sheets with a hydrophobic cavity in the center of its 3D structure, which can accommodate different types of lipid components (45). For example, human MD2 specifically binds to lipid A (the lipid moiety of LPS), which is just located in the hydrophobic pocket of human MD2, thereby participating in immune response against Gram-negative bacteria. *Pj*ML interacts with a lipid component of WSSV envelope *via* its ML domain, participating in antiviral immune responses (28). Besides, a few ML proteins have binding activities to LTA and PGN (31, 46), and another ML protein from a Japanese carpenter ant delivers a variety of hydrophobic semiochemicals involved in chemical communication (47). In the present study, we found that the deep hydrophobic cavity of *Pv*ML1 could accommodate lipid A, and it could also bind to the bacterial surface component LPS by interacting with lipid A. Therefore, *Pv*ML1 can be regarded as an essential pathogen-binding component involved in immune defense against Gram-negative bacteria in shrimp. The specific binding activity of *Pv*ML1 to LPS satisfies one of the two essential requirements for a potential PRR.

In addition to the specific binding ability to pathogens, the association of classical PRRs with pathogens can induce or activate certain immune responses to generate immune effectors for eradicating intruders (48). Classical PRRs are regarded as “switch molecules” in immune signaling pathways, such as PGRPs, which are the key PRRs in the *Drosophila* Toll and IMD signaling pathways (49). The interaction of PGRPs with their specific ligands can activate a series of immune responses to regulate the expression of downstream AMPs (50). In mammals, human MD2 binds LPS and TLR4 to form a ternary complex, and the TLR4 signaling pathway is activated to produce proinflammatory factors against bacterial infection (51). Similarly, *Sp*MD2 specifically binds to LPS and regulates the expression of AMPs, showing remarkable antibacterial activity in mud crab (29). In the present study, we found that *Pv*ML1 could also bind to LPS and participate in antibacterial immune response by affecting the expression of several downstream AMPs. Resembling human MD2, *Sp*MD2 and *Pv*ML1 presented close evolutionary relationships and similar antibacterial activities. We speculate that *Pv*ML1 may act as a potential PRR, similar to human MD2 or *Sp*MD2, for a certain immune signaling pathway against bacterial infection. The aforementioned finding also means that *Pv*ML1 is involved in the activation of a particular immune signaling pathway by regulating the AMP expression. This function meets the second essential requirement for PRRs.

Although both shrimp and fruit fly are arthropods, the Toll and IMD signaling pathways characterized in shrimps seem notably different from the two classical pathways in *Drosophila* (19). As we know, most PGRPs act as key PRRs for Toll and IMD signaling pathways in *Drosophila.* However, no PGRP homolog has been characterized in shrimp or other crustaceans (25–27), although the PGRP family members are abundantly present in insects. We speculate that PGRP homologs may be absent in crustaceans because not a single one has been identified from crustacean species despite the extensive genome and transcriptome data obtained with the help of high-throughput sequencing technologies (25–27). Thus, the recognition and activation mechanism of the Toll signaling pathway in shrimp may be different from that in *Drosophila*, and some other molecules may function in this process. In mammals, with the cooperation of the accessory receptor MD2, human TLR4 mediates the LPS signaling pathway participating in antibacterial infection. Similar recognition and activation mechanisms may be adopted by a Toll signaling pathway in shrimp. Therefore, the similarities between shrimp Tolls and human TLR4 were analyzed to determine whether there is a TLR4 homolog in shrimp. *Pv*Toll1 and *Pv*Toll2 presented high similarities with human TLR4. We also found that the extracellular region of *Pv*Toll2 interacted with the *Pv*ML1-LPS complex to form a ternary complex, and *Pv*ML1 regulated the expression of several AMP genes affecting bacterial infection. In combination with a previous study that showed *Pv*Toll2 significantly activating the promoters of the NF-κB-pathway-controlled AMP genes and mediating the signaling pathway against Gram-negative bacteria (33), we speculate that the LPS–*Pv*ML1–*Pv*Toll2–AMP signaling pathway against Gram-negative bacteria may exist in shrimp. This suggests that *Pv*ML1 may act as a recognition receptor located upstream of the *Pv*Toll2 signal pathway and participate in the bacterial recognition and activation of this pathway.

In this study, we observed that *Pv*ML1 interacted with *Pv*Toll1. In an early report, *Pv*Toll1 was shown that it participated in the anti-*Vibrio* immune response but could not regulate the expression of AMPs (33). Thus, though human Tolls often form homodimers or heterodimers, we conjecture that *Pv*Toll1 may not participate in the LPS–*Pv*ML1–*Pv*Toll2–AMP pathway by forming a heterodimer with *Pv*Toll2, but have other roles in immune system. Actually, *Pv*Toll1 participate in activities involved in cellular immunity (52). In the *Pv*Toll1-knocked-down shrimp, the phagocytotic ability of the hemocytes was significantly decreased. Besides, *Es*ML3, another ML homolog from mitten crab was proved to mediate cellular immunity by promoting phagocytosis of bacteria (30). Based on these findings, we speculate that both *Pv*ML1 and *Pv*Toll1 may be involved in cellular immunity of shrimp, and the interaction between these two molecules may promote the antibacterial immune response. However, more evidence is still required to prove the hypothesis.

The IMD signaling pathway is always regarded as the classical immune process against Gram-negative bacteria in insects. Recent evidence has shown the existence of the IMD signaling pathway in shrimp (53), which suggests that this pathway may play a crucial role in the immune defense against Gram-negative bacteria. However, the presence of the IMD signaling pathway in shrimp does not rule out the existence of the LPS–*Pv*ML1–*Pv*Toll2–AMP signaling pathway. The innate immunity system of arthropods comprises multiple immune strategies to eradicate invading Gram-negative bacteria. In addition to the IMD signaling pathway, a few arthropod LGBPs recognize LPS and activate the prophenoloxidase (PPO) system, thus playing a crucial role in the clearance of Gram-negative bacteria (7, 54). The PPO-activating system is also present in shrimp (55, 56). Thus, the LPS–*Pv*ML1–*Pv*Toll2–AMP pathway may coexist with the IMD and PPO immune routes, forming a more efficient innate immune defense system against Gram-negative bacteria in shrimp.

**Figure 7:**
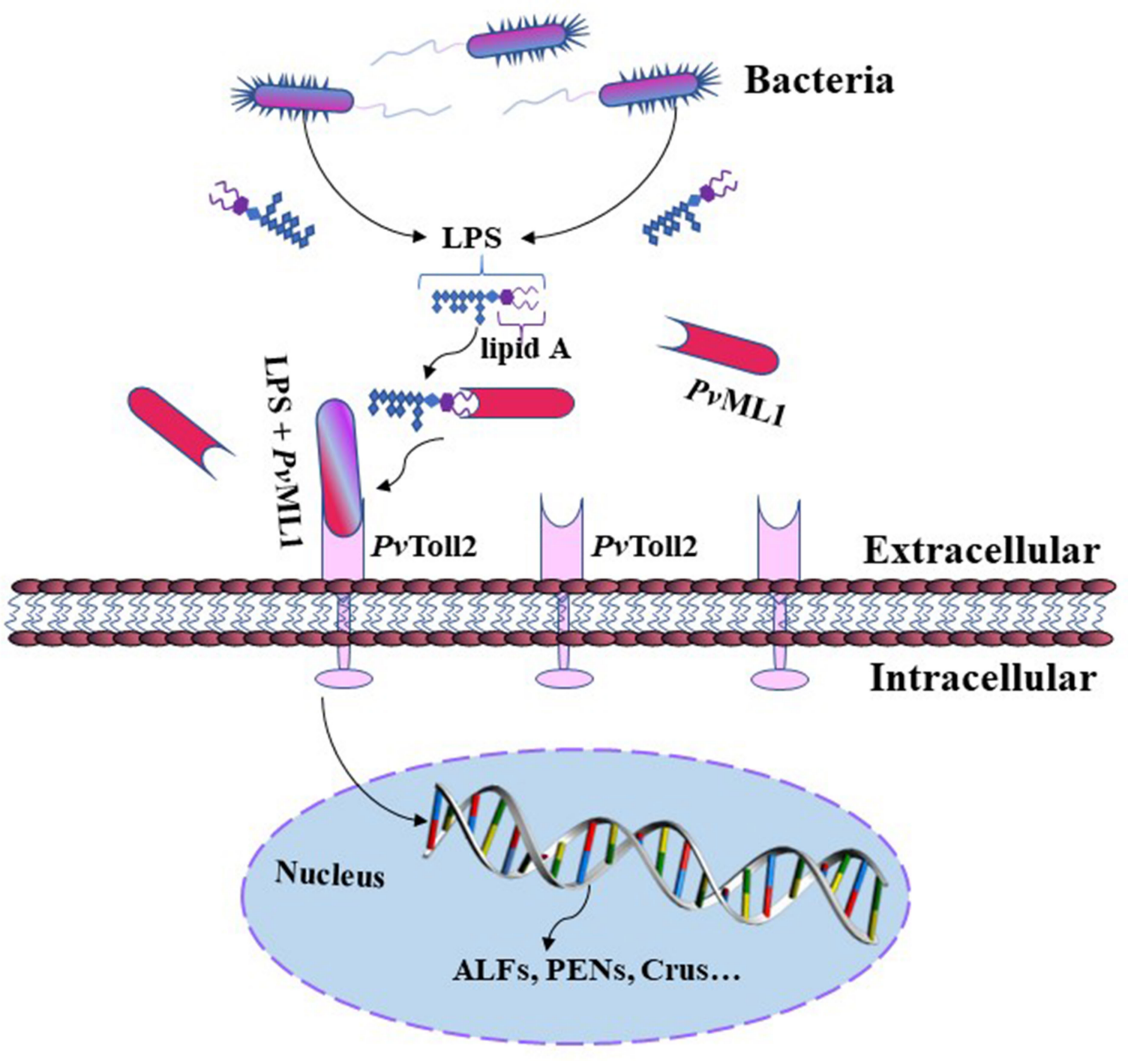
Schematic of the putative antibacterial model mediated by *Pv*ML1. *Pv*ML1 can sense bacterial invasion and bind LPS on the surface of Gram-negative bacterial cells. *Pv*ML1 then interacts with the extracellular region of *Pv*Toll2 forming a ternary complex, which may activate *Pv*Toll2-mediated signaling pathway and induce the expression of downstream specific antimicrobial peptides.

In conclusion, *Pv*ML1, a potential MD2 homolog in shrimp, was characterized in the present study. *Pv*ML1 could recognize the lipid A portion of LPS on Gram-negative cells and specifically interact with *Pv*Toll2, forming a recognition complex. Furthermore, *Pv*ML1 could control bacterial infection by regulating the expression of some AMPs. Thus, a possible antibacterial model mediated by *Pv*ML1 is proposed as follows. *Pv*ML1 can sense the bacterial invasion by binding to their LPS and act as a potential recognition receptor for Gram-negative bacteria; thereafter, the *Pv*Toll2-mediated signaling pathway is activated by the interaction of *Pv*Toll2 with *Pv*ML1 to eliminate the invading bacteria *via* the production of specific AMPs (**Figure 7**). The identification of LPS–*Pv*ML1–*Pv*Toll2–AMP signaling pathway provides new insights into the recognition and activation mechanism of Toll signaling pathways of invertebrates and the defense functions of ML members.

## Data availability statement

The original contributions presented in the study are included in the article/**Supplementary Material**. Further inquiries can be directed to the corresponding authors.

## Author contributions

X-CL and Y-HC conceived and designed the experiments. YW, L-GY and X-CL wrote the manuscript. YW and L-GY conducted most of the experiments. Z-LY, G-PF, S-HL, J-FZ and W-HF contributed experimental suggestions and revised the manuscript. All authors contributed to the article and approved the submitted version.

## References

[1] Yin Q Tian Y Kabaleeswaran V Jiang X Tu D Eck MJ. Cyclic di-GMP sensing *via* the innate immune signaling protein STING. Mol Cell (2012) 46(6):735–45. doi: 10.1016/j.molcel.2012.05.029 PMC3697849 22705373

[2] Das S Nikolaidis N Goto H McCallister C Li J Hirano M. Comparative genomics and evolution of the alpha-defensin multigene family in primates. Mol Biol Evol (2010) 27(10):2333–43. doi: 10.1093/molbev/msq118 PMC2981490 20457584

[3] Akira S Uematsu S Takeuchi O. Pathogen recognition and innate immunity. Cell (2006) 124:783–801. doi: 10.1016/j.cell.2006.02.015 16497588

[4] Akira S Takeda K. Toll-like receptor signalling. Nat Rev Immunol (2004) 4(7):499–511. doi: 10.1038/nri1391 15229469

[5] Gordon S. Pattern recognition receptors: doubling up for the innate immune response. Cell (2002) 111:927–30. doi: 10.1016/S0092-8674(02)01201-1 12507420

[6] Kang D Liu G Lundström A Gelius E Steiner H. A peptidoglycan recognition protein in innate immunity conserved from insects to humans. Proc Natl Acad Sci USA (1998) 95:10078–82. doi: 10.1073/pnas.95.17.10078 PMC21464 9707603

[7] Lee SY Wang R Söderhäll K. A lipopolysaccharide- and beta-1,3-glucan-binding protein from hemocytes of the freshwater crayfish *Pacifastacus leniusculus.* purification, characterization, and cDNA cloning. J Biol Chem (2000) 275(2):1337–43. doi: 10.1074/jbc.275.2.1337 10625682

[8] Inohara N Nuñez G. ML – a conserved domain involved in innate immunity and lipid metabolism. Trends Biochem Sci (2002) 27:219–21. doi: 10.1016/S0968-0004(02)02084-4 12076526

[9] Liao JX Yin ZX Huang XD Weng SP Yu XQ He JG. Cloning and characterization of a shrimp ML superfamily protein. Fish Shellfish Immunol (2011) 30:713–19. doi: 10.1016/j.fsi.2010.12.030 21220027

[10] Jain V Halle A Halmen KA Lien E Charrel-Dennis M Ram S. Phagocytosis and intracellular killing of MD-2 opsonized gram-negative bacteria depend on TLR4 signaling. Blood (2008) 111(9):4637–45. doi: 10.1182/blood-2007-11-126862 18203953 PMC2343597

[11] Ohto U Fukase K Miyake K Shimizu T. Structural basis of species-specific endotoxin sensing by innate immune receptor TLR4/MD-2. Proc Natl Acad Sci USA (2012) 109:7421–26. doi: 10.1073/pnas.1201193109 22532668 PMC3358893

[12] Viriyakosol S Tobias PS Kitchens RL Kirkland TN. MD-2 binds to bacterial lipopolysaccharide. J Biol Chem (2001) 276:38044–51. doi: 10.1074/jbc.M105228200 11500507

[13] West AP Koblansky AA Ghosh S. Recognition and signaling by toll-like receptors. Annu Rev Cell Dev Biol (2006) 22:409–37. doi: 10.1146/annurev.cellbio.21.122303.115827 16822173

[14] Kurata S. Peptidoglycan recognition proteins in drosophila immunity. Dev Comp Immunol (2014) 42:36–41. doi: 10.1016/j.dci.2013.06.006 23796791 PMC3808481

[15] Ip YT Reach M Engstrom Y Kadalayil L Cai H Gonzalez-Crespo S. Dif, a dorsal-related gene that mediates an immune response in *Drosophila* . Cell (1993) 75(4):753–63. doi: 10.1016/0092-8674(93)90495-C 8242747

[16] Nicolas E Reichhart JM Hoffmann JA Lemaitre B. *In vivo* regulation of the IkappaB homologue cactus during the immune response of *Drosophila* . J Biol Chem (1998) 273(17):10463–9. doi: 10.1074/jbc.273.17.10463 9553105

[17] Tauszig-Delamasure S Bilak H Capovilla M Hoffmann JA Imler JL. *Drosophila* MyD88 is required for the response to fungal and gram-positive bacterial infections. Nat Immunol (2002) 3(1):91–7. doi: 10.1038/ni747 11743586

[18] Jin MS Lee JO. Structures of the toll-like receptor family and its ligand complexes. Immunity (2008) 29:182–91. doi: 10.1016/j.immuni.2008.07.007 18701082

[19] Sun JJ Xu S He ZH Shi XZ Zhao XF Wang JX. Activation of toll pathway is different between kuruma shrimp and *Drosophila* . Front Immunol (2017) 8:1151. doi: 10.3389/fimmu.2017.01151 28979261 PMC5611483

[20] Kawai T Akira S. Toll-like receptors and their crosstalk with other innate receptors in infection and immunity. Immunity (2011) 34(5):637–50. doi: 10.1016/j.immuni.2011.05.006 21616434

[21] Liu LK Liu MJ Li DL Liu HP. Recent insights into anti-WSSV immunity in crayfish. Dev Comp Immunol (2021) 116:103947. doi: 10.1016/j.dci.2020.103947 33253753

[22] Wang PH Huang T Zhang X He JG. Antiviral defense in shrimp: from innate immunity to viral infection. Antiviral Res (2014) 108:129–41. doi: 10.1016/j.antiviral.2014.05.013 24886688

[23] Pan G Bao J Ma Z Song Y Han B Ran M. Invertebrate host responses to microsporidia infections. Dev Comp Immunol (2018) 83:104–13. doi: 10.1016/j.dci.2018.02.004 29428490

[24] Takehana A Yano T Mita S Kotani A Oshima Y Kurata S. Peptidoglycan recognition protein (PGRP)-LE and PGRP-LC act synergistically in *Drosophila* immunity. EMBO J (2004) 23(23):4690–700. doi: 10.1038/sj.emboj.7600466 PMC533052 15538387

[25] Qin Z Babu VS Wan Q Zhou M Liang R Muhammad A. Transcriptome analysis of pacific white shrimp (*Litopenaeus vannamei*) challenged by vibrio parahaemolyticus reveals unique immune-related genes. Fish Shellfish Immunol (2018) 77:164–74. doi: 10.1016/j.fsi.2018.03.030 29567139

[26] Zhang X Yuan J Sun Y Li S Gao Y Yu Y. Penaeid shrimp genome provides insights into benthic adaptation and frequent molting. Nat Commun (2019) 10:356. doi: 10.1038/s41467-018-08197-4 30664654 PMC6341167

[27] Zhao M Wang W Zhang F Ma C Liu Z Yang MH. A chromosome-level genome of the mud crab (*Scylla paramamosain* estampador) provides insights into the evolution of chemical and light perception in this crustacean. Mol Ecol Res (2021) 21:1299–317. doi: 10.1111/1755-0998.13332 33464679

[28] Gao J Wang JX Wang XW. MD-2 homologue recognizes the white spot syndrome virus lipid component and induces antiviral molecule expression in shrimp. J Immunol (2019) 203:1131–41. doi: 10.4049/jimmunol.1900268 31331974

[29] Wang Y Zhao S Zhang B Ma HY Fang WH Sheng WQ. A novel ML domain-containing protein (*Sp*MD2) functions as a potential LPS receptor involved in anti-*Vibrio* immune response. Dev Comp Immunol (2020) 103:103529. doi: 10.1016/j.dci.2019.103529 31669309

[30] Song Y Zhou K Nan X Qin Y Zhao K Li W. A novel ML protein functions as a pattern recognition protein in antibacterial responses in *Eriocheir sinensis* . Dev Comp Immunol (2022) 127:104310. doi: 10.1016/j.dci.2021.104310 34762938

[31] Shi XZ Zhong X Yu XQ. *Drosophila melanogaster* NPC2 proteins bind bacterial cell wall components and may function in immune signal pathways. Insect Biochem Mol Biol (2012) 42:545–56. doi: 10.1016/j.ibmb.2012.04.002 PMC3358802 22580186

[32] Zhang R Li X Zhang J Li Y Wang Y Song Y. Toll9 from *Bombyx mori* functions as a pattern recognition receptor that shares features with toll-like receptor 4 from mammals. Proc Natl Acad Sci USA (2021) 118:e2103021118. doi: 10.1073/pnas.2103021118 33963082 PMC8126858

[33] Wang PH Liang JP Gu ZH Wan DH Weng SP Yu XQ. Molecular cloning, characterization and expression analysis of two novel tolls (*Lv*Toll2 and *Lv*Toll3) and three putative spätzle-like toll ligands (*Lv*Spz1-3) from *Litopenaeus vannamei* . Dev Comp Immunol (2012) 36:359–71. doi: 10.1016/j.dci.2011.07.007 21827783

[34] Nielsen H Engelbrecht J Brunak S von Heijne G. Identification of prokaryotic and eukaryotic signal peptides and prediction of their cleavage sites. Protein Eng (1997) 10:1–6. doi: 10.1093/protein/10.1.1 9051728

[35] Kumar S Stecher G Tamura K. MEGA7: Molecular evolutionary genetics analysis version 7.0 for bigger datasets. Mol Biol Evol (2016) 33:1870–4. doi: 10.1093/molbev/msw054 27004904 PMC8210823

[36] Li XC Zhu L Li LG Ren Q Huang YQ Lu JX. A novel myeloid differentiation factor 88 homolog, *Sp*MyD88, exhibiting *Sp*Toll-binding activity in the mud crab *Scylla paramamosain* . Dev Comp Immunol (2013) 39:313–22. doi: 10.1016/j.dci.2012.11.011 23280154

[37] Li H Yin B Wang S Fu Q Xiao B Lǚ K. RNAi screening identifies a new toll from shrimp *Litopenaeus vannamei* that restricts WSSV infection through activating dorsal to induce antimicrobial peptides. PloS Pathog (2018) 14(9):e1007109. doi: 10.1371/journal.ppat.1007109 30256850 PMC6175524

[38] Livak KJ Schmittgen TD. Analysis of relative gene expression data using real-time quantitative PCR and the 2(-delta delta C(T)) method. Methods (2001) 25:402–8. doi: 10.1006/meth.2001.1262 11846609

[39] Wang Y Zhang XW Wang H Fang WH Ma HY Zhang F. *Sp*Crus3 and *Sp*Crus4 share high similarity in mud crab (*Scylla paramamosain*) exhibiting different antibacterial activities. Dev Comp Immunol (2018) 82:139–51. doi: 10.1016/j.dci.2018.01.006 29352984

[40] Du ZQ Wang Y Ma HY Shen XL Wang K Du J. A new crustin homologue (*Sp*Crus6) involved in the antimicrobial and antiviral innate immunity in mud crab, *Scylla paramamosain* . Fish Shellfish Immunol (2019) 84:733–43. doi: 10.1016/j.fsi.2018.10.072 30381264

[41] Zhang XW Wang Y Wang XW Wang L Mu Y Wang JX. A c-type lectin with an immunoglobulin-like domain promotes phagocytosis of hemocytes in crayfish *Procambarus clarkii* . Sci Rep (2016) 6:29924. doi: 10.1038/srep29924 27411341 PMC4944128

[42] Li XC Zhou J Zhou JF Wang Y Ma HY Wang Y. *Sp*Bark suppresses bacterial infection by mediating hemocyte phagocytosis in an invertebrate model, *Scylla paramamosain* . Front Immunol (2019) 10:1992. doi: 10.3389/fimmu.2019.01992 31507600 PMC6716108

[43] Tassanakajon A Amparyup P Somboonwiwat K Supungul P. Cationic antimicrobial peptides in penaeid shrimp. Mar Biotechnol (2011) 13(4):639–57. doi: 10.1007/s10126-011-9381-8 21533916

[44] Waterhouse RM Kriventseva EV Meister S Xi Z Alvarez KS Bartholomay LC. Evolutionary dynamics of immune-related genes and pathways in disease-vector mosquitoes. Science (2007) . 316:1738–43. doi: 10.1126/science.1139862 PMC2042107 17588928

[45] Kim HM Park BS Kim JI Kim SE Lee J Oh SC. Crystal structure of the TLR4-MD-2 complex with bound endotoxin antagonist eritoran. Cell (2007) 130:906–17. doi: 10.1016/j.cell.2007.08.002 17803912

[46] Zhang RN Ren FF Zhou CB Xu JF Yi HY Ye MQ. An ML protein from the silkworm bombyx mori may function as a key accessory protein for lipopolysaccharide signaling. Dev Comp Immunol (2018) 88:94–103. doi: 10.1016/j.dci.2018.07.012 30009928

[47] Ishida Y Tsuchiya W Fujii T Fujimoto Z Miyazawa M Ishibashi J. Niemann-pick type C2 protein mediating chemical communication in the worker ant. Proc Natl Acad Sci USA (2014) 111:3847–52. doi: 10.1073/pnas.1323928111 24567405 PMC3956204

[48] Kawai T Akira S. The roles of TLRs, RLRs and NLRs in pathogen recognition. Int Immunol (2009) 21:317–37. doi: 10.1093/intimm/dxp017 PMC2721684 19246554

[49] Mellroth P Karlsson J Håkansson J Schultz N Goldman WE Steiner H. Ligand-induced dimerization of *Drosophila* peptidoglycan recognition proteins in vitro. Proc Natl Acad Sci USA (2005) 102(18):6455–60. doi: 10.1073/pnas.0407559102 15843462 PMC1088352

[50] Wang Q Ren M Liu X Xia H Chen K. Peptidoglycan recognition proteins in insect immunity. Mol Immunol (2019) 106:69–76. doi: 10.1016/j.molimm.2018.12.021 30590209

[51] Ryu JK Kim SJ Rah SH Kang JI Jung HE Lee D. Reconstruction of LPS transfer cascade reveals structural determinants within LBP, CD14, and TLR4-MD2 for efficient LPS recognition and transfer. Immunity (2017) 46:38–50. doi: 10.1016/j.immuni.2016.11.007 27986454

[52] Han-Ching Wang K Tseng CW Lin HY Chen IT Chen YH Chen YM. RNAi knock-down of the *Litopenaeus vannamei* toll gene (LvToll) significantly increases mortality and reduces bacterial clearance after challenge with *Vibrio harveyi* . Dev Comp Immunol (2010) 34(1):49–58. doi: 10.1016/j.dci.2009.08.003 19698743

[53] Li F Xiang J. Signaling pathways regulating innate immune responses in shrimp. Fish Shellfish Immunol (2013) 34(4):973–80. doi: 10.1016/j.fsi.2012.08.023 22967763

[54] Amparyup P Sutthangkul J Charoensapsri W Tassanakajon A. Pattern recognition protein binds to lipopolysaccharide and β-1,3-glucan and activates shrimp prophenoloxidase system. J Biol Chem (2012) 287(13):10060–9. doi: 10.1074/jbc.M111.294744 PMC3322982 22235126

[55] Tassanakajon A Rimphanitchayakit V Visetnan S Amparyup P Somboonwiwat K Charoensapsri W. Shrimp humoral responses against pathogens: antimicrobial peptides and melanization. Dev Comp Immunol (2018) 80:81–93. doi: 10.1016/j.dci.2017.05.009 28501515

[56] Chen YY Chen JC Kuo YH Lin YC Chang YH Gong HY. Lipopolysaccharide and β-1,3-glucan-binding protein (LGBP) bind to seaweed polysaccharides and activate the prophenoloxidase system in white shrimp *Litopenaeus vannamei* . Dev Comp Immunol (2016) 55:144–51. doi: 10.1016/j.dci.2015.10.023 26522339

