# Supplementary material for: PvML1 suppresses bacterial infection by recognizing LPS and regulating AMP expression in shrimp
Source: Front Immunol. 2022 Dec 28;13:1088862. doi: 10.3389/fimmu.2022.1088862 (PMC9832027; doi:10.3389/fimmu.2022.1088862)
Supplement: Supplementary file 1 [file DataSheet_1.docx]

1 GCGGGGACGAGGCGGCAGCGCCCAGCCCCACCGGCGGGCACACTTAAAAGTAGTTGGGGATGGCGAGGACGAGGCAGTGTGAGCTTAGCC

91 GTCGTCAGGACTCGGAGTGTGATTAGCCCGCGTGCGTGTAGTGTGTAGTATACCATGGCTGCCTCTTCCTCTTGTCTCTTCGTCGCCCTC

1 M A A S S S C L F V A L

181 GCCCTCTGCCTCGTCGGGTCGTCGTGGGGGGAGGTGCACGAGATCCCCGTCAGGAGCTGTGATGGAGCCACAGCGCCAAGCAGCCTGAAG

13 A L C L V G S S W G E V H E I P V R S **C** D G A T A P S S L K

271 ATGGAATGCACACAGTATCTCCGTGGGACTTGCATACTCGAGAAAGGGCAAACATACAACCTCACAGCTGAGTTCACGCCAGACCGAAAC

43 M E **C** T Q Y L R G T **C** I L E K G Q T Y N L T A E F T P D R N

361 CTCCGGGAGGTGAAGAGCCACGCAGCCTGGAAGACGTGGGTCGAGATGCCCCTGTACGGGCAGGAGAGCCAAGTCTGCAACGGCGTGTAC

73 L R E V K S H A A W K T W V E M P L Y G Q E S Q V **C** N G V Y

451 CTGACCTGCCCTTTGCGCTCAGGTCAGCCAACGAGGTTCTCATACCCGTTCCACGTCCACGAGTTCCTGATGAGGCGGCGATACCCCGTC

103 L T **C** P L R S G Q P T R F S Y P F H V H E F L M R R R Y P V

541 ATCTGGCGACTGAGGGACGTGGACAGCGAAGACACAACGCTGTGTTTCGTCTTCAATGTTAAAATCTTGTAAGGAAAACACACACACGCA

133 I W R L R D V D S E D T T L **C** F V F N V K I L *

631 CACGCACACACACACACACACA

**FIGURE S1.** The cDNA sequence and deduced amino acids of *Pv*ML1 from *Penaeus vannamei*. Signal peptide was shown in red letters, MD-2-related lipid-recognition (ML) domain was shade and the stop codon was indicated by an asterisk (*). The conserved cysteine residues were shown in bold and blue.
